# Self-Nanoemulsifying Drug Delivery System for Resveratrol: Enhanced Oral Bioavailability and Reduced Physical Fatigue in Rats

**DOI:** 10.3390/ijms18091853

**Published:** 2017-08-25

**Authors:** Ching-Chi Yen, Chih-Wei Chang, Mei-Chich Hsu, Yu-Tse Wu

**Affiliations:** 1 School of Pharmacy, College of Pharmacy, Kaohsiung Medical University, 100, Shih-Chuan 1st Rd., Kaohsiung 80708, Taiwan; date0315@hotmail.com (C.-C.Y.); wxes9050304@gmail.com (C.-W.C.); 2 Department of Sports Medicine, Kaohsiung Medical University, 100, Shih-Chuan 1st Rd., Kaohsiung 80708, Taiwan; 3 Department of Medical Research, College of Medicine, Kaohsiung Medical University Hospital, 100, Tzyou 1st Rd., Kaohsiung 80708, Taiwan

**Keywords:** resveratrol, self-nanoemulsifying drug delivery system, anti-fatigue

## Abstract

Resveratrol (RES), a natural polyphenolic compound, exerts anti-fatigue activity, but its administration is complicated by its low water solubility. To improve RES bioavailability, this study developed a self-nanoemulsifying drug delivery system (SNEDDS) for RES and evaluated its anti-fatigue activity and rat exercise performance by measuring fatigue-related parameters, namely lactate, ammonia, plasma creatinine phosphokinase, and glucose levels and the swimming time to exhaustion. Through solubility and emulsification testing, the optimized SNEDDS composed of Capryol 90, Cremophor EL, and Tween 20 was developed; the average particle size in this formulation, which had favorable self-emulsification ability, was approximately 41.3 ± 4.1 nm. Pharmacokinetic studies revealed that the oral bioavailability of the optimized RES-SNEDDS increased by 3.2-fold compared with that of the unformulated RES-solution. Pretreatment using the RES-SNEDDS before exercise accelerated the recovery of lactate after exercise; compared with the vehicle group, the plasma ammonia level in the RES-SNEDDS group significantly decreased by 65.4%, whereas the glucose level significantly increased by approximately 1.8-fold. Moreover, the swimming time to exhaustion increased by 2.1- and 1.8-fold, respectively, compared with the vehicle and RES-solution pretreatment groups. Therefore, the developed RES-SNEDDS not only enhances the oral bioavailability of RES but may also exert anti-fatigue pharmacological effect.

## 1. Introduction

Fatigue is defined as physical and/or mental weariness resulting in inability to maintain exercise at the same intensity, leading to deterioration in performance [1]. Oxidative stress and exhaustion are two possible influential mechanisms in physical fatigue [2]. The accumulation of reactive oxygen species (ROS) induces oxidative stress in the body and may cause physical injury by attacking large molecules and cell organs, resulting in physical fatigue. Exhaustion, defined as energy depletion and excess metabolite accumulation, may play a crucial role in physical fatigue [3]. Recent studies have investigated supplementing exogenous antioxidants, such as icariin [4] and (−)-epigallocatechin-3-gallate [5], through diet to prevent exercise-induced oxidative stress and reduce physical fatigue by scavenging free radicals and ROS. In particular, resveratrol (RES), an antioxidant, has been widely utilized to enhance exercise performance [6,7]. RES, in combination with exercise, can clinically enhance the mitochondrial capacity of the forearm skeletal muscle [8]. Some researchers have studied the anti-fatigue effects of RES. For example, Xiao reported that gavaging 25 mg/kg RES once daily for four weeks significantly prolonged the exercise time to exhaustion [9]. Wu et al. demonstrated that giving RES once daily for four weeks exerted anti-fatigue pharmacological effects [10]. In addition, Menzies et al. showed that RES treatment for nine weeks combined with exercise training could stimulate mitochondrial biogenesis [11]. Although these anti-fatigue effects of RES are positive, the pretreatment protocols are time-consuming, possibly due to the low bioavailability of RES.

RES (*trans*-3,5,4′-trihydroxystilbene) is a phytoalexin present in red wine, some grapes and berries, and various herbs. Apart from its anti-physical-fatigue effects [10], RES has demonstrated extensive bioactivities, such as anti-inflammatory [12], cardioprotective [13], and cancer prevention [14] activities. Although RES has numerous health benefits, its therapeutic use has been hindered by its low aqueous solubility and dissolution rate, which reduce its oral bioavailability. Furthermore, the in vivo biological effects of RES appear to be strongly limited by its low bioavailability. Low solubility and high permeability are typical of class II compounds [15,16], of which RES is one; consequently, they often exhibit solubility-limited absorption, leading to low absorption of the active ingredient. In addition, RES is rapidly metabolized in vivo [17]. Walle et al. reported that after administering a single 25-mg oral dose in humans, only trace amounts of unchanged RES (<5 ng/mL) were detected in plasma [18].

Many strategies have been applied to enhance RES solubility and oral absorption. The proliposomal formulation prepared by Basavaraj et al. significantly improved the absorption rate of unmetabolized RES [19]. Teskač et al. demonstrated the cell uptake of RES-loaded solid lipid nanoparticles, which enhanced the effects of RES on cellular fate [20]. Sessa et al. hypothesized that encapsulation of RES in the inner core of nanoemulsions could limit chemical degradation, consequently improving transport through the cell monolayer [21]. Of the various pharmaceutical dosage forms, self-nanoemulsifying drug delivery system (SNEDDS) is ideal for delivering lipophilic substances. SNEDDS is a colloidal dispersion consisting of oils and surfactants that emulsify under gentle agitation, similar to the conditions encountered in the gastrointestinal tract (GIT) [22], forming oil-in-water nanoemulsions with nanodroplets [23]. SNEDDS is characterized by their excellent stability, which helps overcome the stability problem of solid lipid nanoparticles and liposomes, and their higher ease of manufacture relative to emulsions. In addition, SNEDDS can improve the rate and extent of absorption, thus enhancing the bioavailability of lipophilic drugs. Moreover, interactions between certain excipients (e.g., Cremophor, Solutol HS-15, Tween 20, Tween 80, Labrasol, and vitamin E-TPG) and enzymes or transporters have raised considerable academic interest regarding the effects of such systems on drug absorption and metabolism [24], and significant improvements in the oral bioavailability of these drug compounds have been demonstrated. Although some previous studies have proposed the use of SNEDDS for RES, the lack of pharmacokinetic data of these studies could hardly confirm the absorption effect of RES [25,26,27]. Only one study has accessed the oral bioavailability of the developed RES-SNEDDS [28]. In this study, we focused on the alleviation of physical fatigue, which is an important issue in the field of sports medicine. Therefore, we hypothesized that the use of nano-formulation might improve the oral bioavailability and anti-fatigue efficiency of RES. The purpose of this study was to develop and characterize a RES-SNEDDS in order to enhance RES solubility and further improve RES oral bioavailability. In addition, this study investigated whether pretreatment with a single dose of the developed RES-SNEDDS enhances anti-fatigue effects and exercise performance in rats.

## 2. Results and Discussion

### 2.1. Characterization and Optimization of the RES-SNEDDS

**Figure 1:**
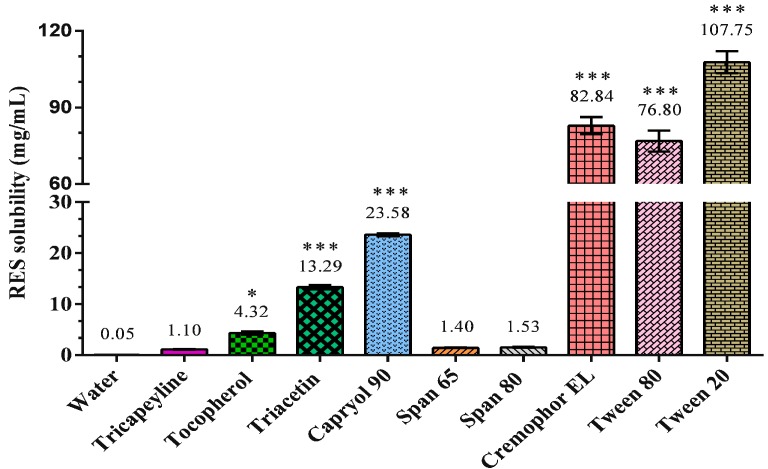
Solubility of resveratrol (RES) in different solvents. Results were expressed as mean ± standard error of the mean (*n* = 3 for each group). * *p* < 0.05 compared with water group. *** *p* < 0.001 compared with water group.

The solubility of RES in various carriers was investigated, and Capryol 90 was found to have the highest RES solubility of 23.58 mg/mL among all oils (Figure 1). Among the investigated surfactants, Cremophor EL and Tween 20 exhibited high RES solubilities of 82.84 ± 1.94 and 107.75 ± 2.50 mg/mL, respectively. Because RES is practically insoluble in water [15], the excipient for the SNEDDS must be selected through solubility studies. We chose Capryol 90 as the oil phase and Cremophor EL and Tween 20 as the surfactants for the SNEDDS formulation.

**Figure 2:**
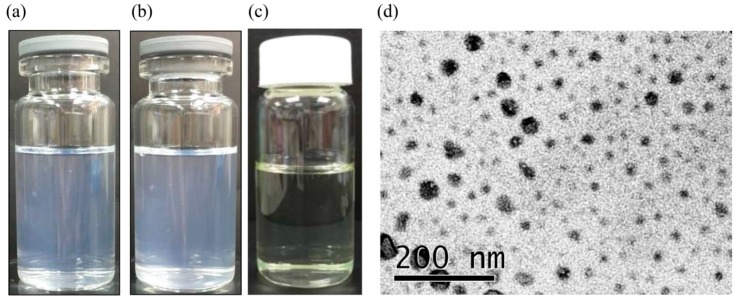
Resveratrol self-nanoemulsifying drug delivery system (RES-SNEDDS) dispersed in: (**a**) water; and (**b**) 0.1 N HCl; (**c**) the appearance of F7 RES-SNEDDS; and (**d**) transmission electron micrograph of F7 RES-SNEDDS.

The self-emulsification efficiency of the RES-SNEDDS was evaluated through dispersibility testing. Only formulation 7 (F7) and 8 (F8) were found to be of grade A (Table 1); that is, capable of rapidly emulsifying within the GIT fluid (Figure 2a,b). In dispersion testing, no differences were observed when purified water or 0.1 N HCl was used as the dispersion medium, and both media could stimulate SNEDDS emulsification in the GIT after oral ingestion. By contrast, formulations involving other dispersion media either required longer emulsification time or did not emulsify at all.

The formulations were diluted with water to measure percentage transmittance and particle size. When transmittance is close to 100%, the formulation is clear and more transparent and its absorbability within the GIT is high. F7 had higher percentage transmittance than did F8, indicating that F7 might have relatively higher absorbability in the GIT. Therefore, we used F7 (Figure 2c) for the subsequent experiments in this study. The particle size of all formulations was less than 100 nm; F7 exhibited a unimodal particle-size distribution pealing at 41.3 ± 4.1 nm with a polydispersity index of 0.38 ± 0.12, and the droplet size of F7 was verified through transmission electron microscopy (TEM), which indicated a droplet size of approximately 50 nm (Figure 2d).

### 2.2. Pharmacokinetic Study

**Figure 3:**
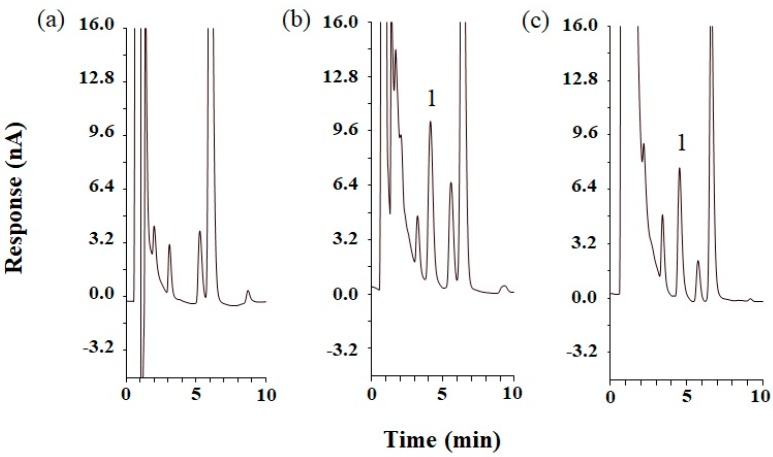
High performance liquid chromatograms of: (**a**) blank rat plasma; (**b**) standard resveratrol (RES) (1000 ng/mL) spiked with blank rat plasma; and (**c**) rat plasma sample at 30 min after oral administration of 50 mg/kg RES self-nanoemulsifying drug delivery system. Peak identification: (1) RES.

**Figure 4:**
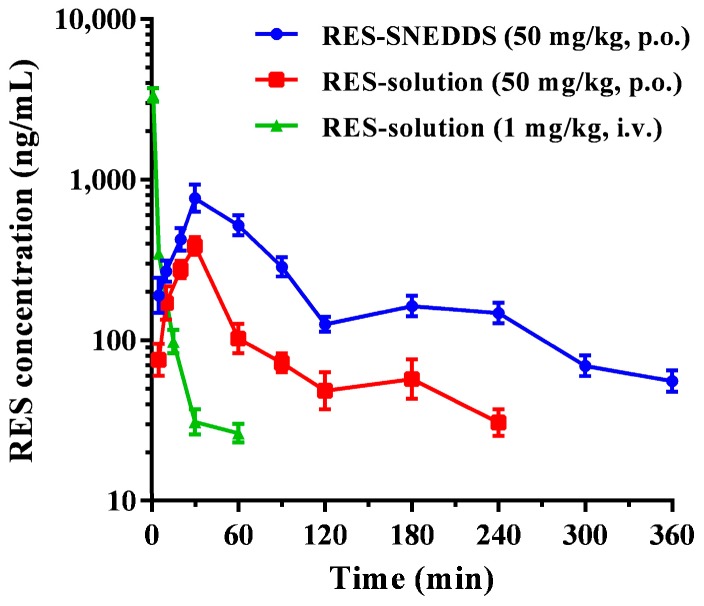
The plasma concentrations-time profiles of resveratrol (RES) after drug administration in rats: (●) indicates the optimal RES self-nanoemulsifying drug delivery system (RES-SNEDDS) (50 mg/kg, p.o.); (■) indicates RES-solution (50 mg/kg, p.o.); and (▲) indicates RES-solution (1 mg/kg, i.v.). Data are expressed as mean ± standard error of the mean (*n* = 5 for each group).

The optimized RES-SNEDDS (F7) was subject to pharmacokinetic studies. Figure 3 presents the representative chromatograms of rat blank plasma, rat blank plasma spiked with standard RES, and plasma sample obtained at 30 min after the oral administration of 50 mg/kg RES-SNEDDS. Figure 4 presents the concentration–time curves of RES in rat blood after the oral administration of the RES-SNEDDS (50 mg/kg, p.o.) and RES-solution (50 mg/kg, p.o.) and those after the single intravenous administration of RES-solution (1 mg/kg, i.v.). The pharmacokinetic parameters are illustrated in Table 2. After oral administration, the mean maximum concentration (*C*_max_) of the optimized RES-SNEDDS was 2.2-fold higher than that of RES-solution (869.2 ± 112.2 and 386.2 ± 68.4 ng/mL, respectively). The elimination half-life (*t*_1/2_) did not differ significantly between RES-SNEDDS and RES-solution groups. The area under curve (AUC) in the optimized RES-SNEDDS and RES-solution were 77,055.4 ± 4857.7 and 23,950.5 ± 3691.3 ng min/mL (50 mg/kg, p.o.), respectively. The oral bioavailability of the RES-SNEDDS was 9.5 ± 1.5%, which was significantly higher than that of the RES-solution group (3.0 ± 0.8%).

The extremely low oral bioavailability of RES is primarily due to its low solubility and extensive intestinal first-pass metabolism [17]. In this study, the low solubility of RES was improved, as evidenced by solubility test results. In addition, the developed SNEDDS improved the oral bioavailability of RES. Previous studies have also reported that such a system for delivering hydrophobic compounds could be an effective oral dosage form for enhancing oral bioavailability [29,30]. Many studies have shown that reducing the particle size increases the absorption of the active ingredient and the particle uptake by enhancing the mechanisms of passive transport through the intestinal walls [31]. Under the gentle digestive motility in the GIT, SNEDDS self-emulsify and rapidly present the drug as small droplets <100 nm in size in the aqueous contents of the stomach [22]. Moreover, the lymphatic transport substantially contributes to the total oral absorption of drugs because the oil phase, which promotes lipophilic drug absorption, constitutes more than 25% of the self-emulsifying drug delivery system [32]. In addition, SNEDDS can protect drugs against enzymatic degradation in the GIT, such as quercetin, luteolin, and epigallocatechin gallate, thus enhancing the stability of the active ingredient [33,34].

The efficiency of self-nanoemulsification is strongly related to the hydrophilic–lipophilic balance (HLB) of surfactants. In this study, because of the high HLB of Cremophor EL and Tween 20, uniform nanoemulsion droplets formed easily within the GIT. Pharmaceutical excipients are generally considered pharmacologically inert; nevertheless, the usage of surfactants may increase permeability by interfering with the lipid bilayer of the epithelial cell membrane. Moreover, efflux system (e.g., P-glycoprotein) and cytochrome P450 (CYP) metabolizing system are major physiological hurdles for the bioavailability of many orally administered drugs. The modulation of efflux transports by Cremophor EL was reported [35]. In addition, according to a previous study, Cremophor EL could competitively inhibit the formation of extensive presystemic glucuronidation metabolites of raloxifene in a human liver microsome experimental model [36]. Thus, the usage of Cremophor EL might also modulate in vivo metabolism of RES to enhance its oral absorption. The optimized RES-SNEDDS in this study improved oral bioavailability by 3.2-fold relative to that of the unformulated RES-solution; this result is attributable to the increase in the solubility of RES and the reduction in its particle size, which in turn increases the surface area for drug absorption [37]. These results evidenced that the developed SNEDDS is an efficient strategy of improving RES oral bioavailability.

### 2.3. Lactate Production and Clearance during High-Intensity Swimming

**Figure 5:**
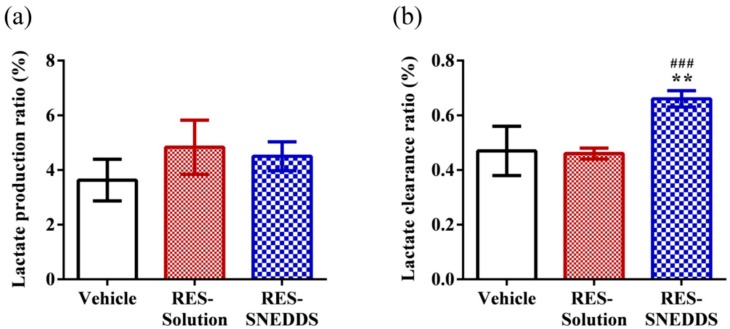
Effect of resveratrol (RES) on: (**a**) lactate production ratio; and (**b**) lactate clearance ratio. Data are expressed as mean ± standard error of the mean (*n* = 5 for each group). ** *p* < 0.01 compared with vehicle group. **^###^** *p* < 0.001 compared with RES-solution group. Lactate production ratio = (Lactate_post-swimming_ − Lactate_pre-swimming_)/Lactate_pre-swimming_; Lactate clearance ratio = (Lactate_post-swimming_ − Lactate_30 min later after post-swimming_)/Lactate_post-swimming_.

The lactate production ratio in the vehicle, RES-solution, and RES-SNEDDS was 3.63 ± 1.87, 4.83 ± 2.42, and 4.50 ± 1.30, respectively (Figure 5a), and the corresponding lactate clearance ratio was 0.47 ± 0.09 0.46 ± 0.05, and 0.66 ± 0.06 (Figure 5b). The relationships between lactate accumulation and work intensity and capacity as well as the correlation between lactate accumulation and muscular fatigue have long been a major research topic [38]. During high-intensity exercise, the muscles produce large quantities of lactate through anaerobic glycolysis-induced energy production. A high lactate level reduces the pH, which in turn induces various biochemical and physiological side effect [39].

In this study, the lactate production ratios of the vehicle, RES-solution, and RES-SNEDDS did not differ significantly; however, the lactate clearance ratio of RES-SNEDDS decreased significantly (by 40.4%) compared with that of the vehicle, whereas the lactate clearance ratio of the RES-solution did not significantly differ from that of the vehicle. Supplementation with certain antioxidant nutrients has previously been demonstrated to be a practical approach for rapid recovery from fatigue and for preventing exercise-induced oxidative damage [40]. Accordingly, we hypothesized that pretreating rats with the optimized RES-SNEDDS before exercise would accelerate recovery from lactate accumulation.

### 2.4. Blood Biochemical Variables after Swimming

**Figure 6:**
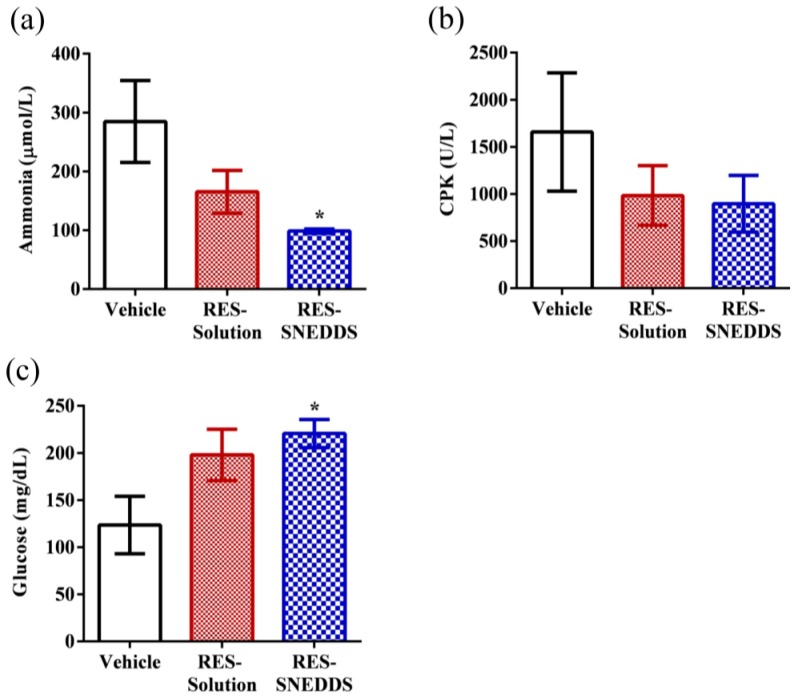
Effect of resveratrol (RES) on: serum (**a**) ammonia; (**b**) plasma creatinine phosphokinase (CPK); and (**c**) glucose levels, after swimming challenge. Data are expressed as mean ± standard error of the mean (*n* = 5 for each group). * *p* < 0.05 compared with vehicle group.

The plasma ammonia level in the vehicle, RES-solution, and RES-SNEDDS was 284.8 ± 69.7, 165.4 ± 36.4, and 98.6 ± 3.6 µmol/L, respectively (Figure 6a). Ammonia is a major protein and an amino-acids metabolite. During exercise, the muscles rapidly produce ammonia because of adenosine monophosphate deamination [41]; this increase in ammonia during exercise has been linked to peripheral and central fatigue [42]. In rats pretreated with the RES-SNEDDS before exercise, the plasma ammonia level decreased significantly (by 65.4%) compared with that in the vehicle group, whereas no significant differences were observed between the RES-solution and vehicle groups. The plasma creatinine phosphokinase (CPK) level in the vehicle, RES-solution, and RES-SNEDDS was 1658.2 ± 627.3, 983.4 ± 316.5, and 895.6 ± 300.7 U/L, respectively (Figure 6b). CPK is a clinical biomarker for muscle damage [43]. The result indicated that CPK in the RES-SNEDDS decreased by 46.0% compared with that in the vehicle group; however, CPK did not differ in both groups because of the large standard error of the mean.

The glucose level is a major index for performance maintenance during exercise, because the energy required for exercise is initially sourced from glycogen decomposition [44]. The glucose level in the vehicle, RES-solution, and RES-SNEDDS was 123.6 ± 30.5, 198.0 ± 27.2, and 220.6 ± 14.9 mg/dL, respectively (Figure 6c). The glucose level in the RES-SNEDDS group was significantly (approximately 1.8-fold) higher than that in the vehicle group, whereas the glucose levels did not differ significantly between the RES-solution and vehicle groups. RES functions, in part, as an allosteric activator of SIRT1, which directly activates FOXO1 in hepatocytes to shift glucose metabolism to gluconeogenesis, thereby promoting glucose release [45,46]; this phenomenon may explain the enhanced blood glucose levels in the RES-SNEDDS group.

### 2.5. Exhaustive Swimming Test

**Figure 7:**
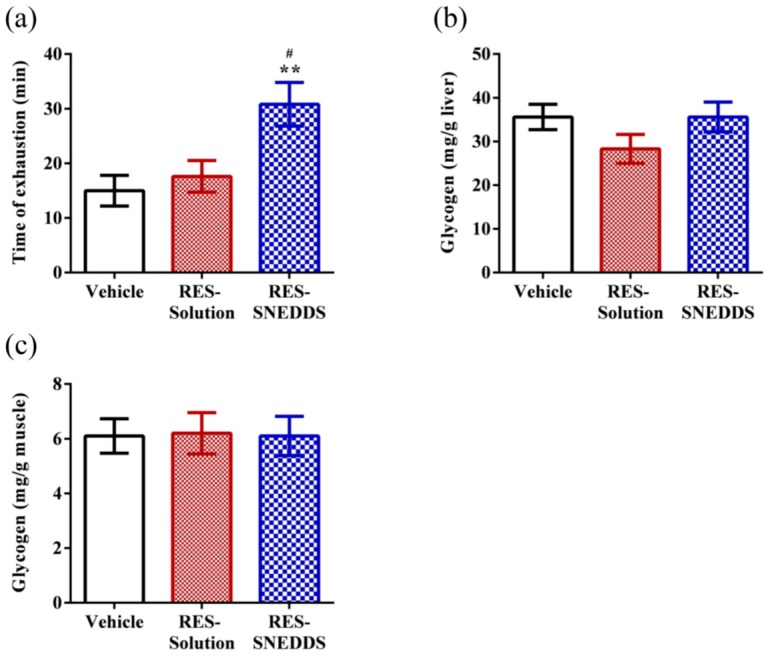
Effect of resveratrol (RES) on: (**a**) exhaustive swimming test; and glycogen content in: (**b**) liver; and (**c**) muscle. Data are expressed as mean ± standard error of the mean (*n* = 5 for each group). ** *p* < 0.01 compared with vehicle group. ^#^ *p* < 0.05 compared with RES-solution group.

The exercise endurance levels of rats administered the vehicle, RES-solution, and RES-SNEDDS was 15.0 ± 2.8, 17.6 ± 2.9, and 30.8 ± 4.0 min, respectively (Figure 7a). Exercise endurance is a major variable in evaluating anti-fatigue treatment. According to our data, pretreatment with RES-solution did not increase the swimming time to exhaustion, which might be due to the low RES absorption. By contrast, pretreatment with the RES-SNEDDS increased the swimming time to exhaustion by 2.1- and 1.8-fold compared with the vehicle and RES-solution groups, respectively, confirming that the RES-SNEDDS improves not only RES bioavailability but also the exercise performance of rats.

### 2.6. Tissue Glycogen Determination

Carbohydrates and catabolized fat are the two major sources of energy during the exercise [47]. Thus, the glucose storage ability of the liver and muscles strongly influence exercise endurance. Glycogen is the main storage form of glucose in vivo. The exhaustive swimming test revealed that the liver and muscle glycogen contents did not differ significantly among the three studied groups, which is consistent with the results of a previous study; this is because RES reduces PGC-1α, a phenomenon likely triggered by SIRT1 activation. SIRT1-induced PGC-1α activation in hepatocytes, can result in downregulation of glycolytic pathways and the upregulation of gluconeogenic pathways to maintain the glycogen level [10], although the plasma glucose level increased (glycolysis process) during exercise (Figure 6c). Moreover, PGC-1α regulates fuel utilization in muscle cells by increasing fatty acid oxidation and shutting down glucose oxidation. In low-glucose conditions, SIRT1 enhances the oxidation of the fatty acid instead of the glycogen to obtain energy in skeletal muscles [48]. These behaviors might explain the improved exercise performance in the RES-SNEDDS group despite only non-significant changes in the liver and muscle glycogen contents among the studied groups (Figure 7b,c).

## 3. Materials and Methods

### 3.1. Materials

RES (purity > 98%), Span 80, Tween 80, and Tween 20 were obtained from Tokyo Chemical Industry (Tokyo, Japan). Capryol 90 (propylene glycol monocaprylate) was purchased from Gattefosse (Saint-Priest Cedex, France). Tricaprylin (glycerol trioctanoate), Cremophor EL (polyoxyl 35 hydrogenated castor oil) and α-tocopherol were supplied by Sigma-Aldrich (St. Louis, MO, USA). Triacetin (glyceryl triacetate) was obtained from Alfa Aesar (Ward Hill, MA, USA). Span 65 was purchased from Merck (Darmstadt, Germany). Acetonitrile for HPLC were obtained from Tedia (Fairfield, OH, USA).

### 3.2. Preparation of RES-SNEDDS

The solubility of RES in different types of oils and emulsifiers were determined. An excess of RES powder was added into each vehicle followed by vortex mixing for 30 s. Then, the mixture was shaken at 37 °C in a water bath for 48 h and centrifuged at 10,000 rpm for 15 min (Mikro 22R, Hettich Zentrifugen, Tuttlingen, Germany) to separate the undissolved RES. The clear supernatant was diluted with appropriate 50% (*v*/*v*) methanol and measured spectrophotometrically at 310 nm (U-5100, Hitachi, Tokyo, Japan). The calibration cure of RES solution, constructed by UV-visible spectrophotometer (U-5100, Hitachi) at 310 nm, was in the range of 1–25 µg/mL.

The RES-SNEDDS preparation procedures were modified from a previous report [49]. Briefly, in the dark environment, RES (120 mg) was dissolved in 12 grams of oil phase (Capryol 90) by magnetic stirring to completely dissolve. The mixture of surfactant (Cremophor EL) and co-surfactant (Tween 20) were added in dropwise to prepare of a total weight of 20 grams and the resulting mixture was stirred for 30 min. Different ratios of oil phase, surfactants were prepared and examined to fine an optimal RES-SNEDDS. The formulation components of RES-SNEDDS were listed in Table 1.

### 3.3. Dispersibility Test and Percentage Transmittance of RES-SNEDDS

Dispersibility test was performed to assess RES-SNEDDS efficiency of self emulsification. An aliquot of 1-mL each formulation was added to 500 mL of 0.1 N HCl or purified water at 37 ± 0.5 °C using a standard US Pharmacopeia XXII dissolution apparatus with paddle rotating at 50 rpm provides gentle agitation [50]. In vitro performance of the formulations were visually assessed using the following grading system [51]: Grade A: Denoting a rapidly forming (within 1 min) emulsion which was clear or slightly bluish in appearance; Grade B: Denoting a rapidly forming, slightly less clear emulsion which had a bluish white appearance; Grade C: Denoting a bright white emulsion (similar in appearance to milk) that formed within 2 min; Grade D: Denoting a dull, greyish white emulsion with a slightly oily appearance that was slow to emulsify (longer than 2 min); Grade E: Denoting a formulation which exhibited either poor or minimal emulsification with large oil droplets present on the surface. Percentage transmittance was assessed using UV-visible spectrophotometer (U-5100, Hitachi). Using double distilled water to dilute the formulation 100 times and analyzed at 500 nm by using double distilled water as blank [52].

### 3.4. Morphological Characterization and Particle Sizing

TEM analysis was used to determine the morphology of RES-SNEDDS. The TEM sample of the selected RES-SNEDDS was diluted and placed on a 300 mesh copper grid coated with carbon. Sample was negatively stained by using a 2% (*w*/*v*) phosphortungsten acid (PTA) solution and removed the excess PTA. The dried grid was examined under TEM (JEM-2100, JEOL, Tokyo, Japan) at a voltage of 200 kV. Approximately 50 mg of the RES-SNEDDS was diluted in a 50 mL volumetric flask by double distilled water (or 0.1 N HCl) before determining the droplet size. Photon Correlation Spectroscopy (Beckman Coulter N5) at an angle of 90° was applied to determine the droplet size and polydispersity index of the RES-SNEDDS at room temperature. Each sample was carried out in triplicate.

### 3.5. Animals and Pharmacokinetic Studies

Animal experimental protocols were reviewed and approved by the Institutional Animal Care and Use Committee (IACUC-104042, approval date: 13 July 2015) of Kaohsiung Medical University Hospital (Kaohsiung, Taiwan). Sprague-Dawley rats weighing 250 ± 50 g were used for the study, which were obtained from BioLASCO Taiwan Co., Ltd. (Taipei, Taiwan). Rats were randomly assigned into three groups (*n* = 5 for each group). For the intravenous group, each rat was given the RES-solution (Dose: 1 mg/kg) through the femoral vein. Blood samples were collected as blank plasma before drug administration, and further blood samples were collected at 1, 5, 15, 30, and 60 min after administration. For the oral group, RES-SNEDDS were administered by gastric gavage (Dose: 50 mg/kg) and RES-solution (Dose: 50 mg/kg) which suspended in 0.25% (*w*/*v*) carboxyl methyl cellulose aqueous solution was used to be control group. Blood samples were collected at 5, 10, 20, 30, 60, 90, 120, 180, 240, 300 and 360 min after administration. An aliquot of 300 µL blood plasma was withdrew manually via the jugular vein catheter and placed into a vial rinsed with heparin. Each blood plasma was centrifuged at 3000 rpm for 10 min and plasma fraction was separated and directly precipitated before HPLC analysis. Briefly, An aliquot of 100 µL plasma were mixed with an equal volume mixture of acetonitrile and 6% (*w*/*v*) perchloric acid (60:40, *v*/*v*), and then vortex-mixed for 1 min to precipitate protein molecules. The supernatant was collected after centrifugation at 10,000 rpm for 10 min and was then filtered through a 0.22-µm syringe filter prior to HPLC analysis.

### 3.6. Analysis of RES by HPLC

The HPLC system consisted of a chromatographic pump (5160), a sample injector (5260) (Hitachi). A Kinetex XB-C18 column (100 mm × 2.1 mm i.d., 2.6 µm, Phenomenex, Torrance, CA, USA), protected by a guard column was used for sample separation. The mobile phase consisted of acetonitrile-0.01 M Na_2_HPO_4_ (23:77, *v*/*v*, pH 2.5 adjusted by orthophosphoric acid) was filtered through a membrane filter (0.45 µm, Merck Millipore, Tullagreen, Carrigtwohill, Ireland) and sonicated (Branson, CT, USA) before used. The flow rate of the mobile phase was 0.4 mL/min and the volume of injection was 20 μL. Electrochemical detection was performed by using an amperometric detector (BAS LC-4C, West Lafayette, IN, USA) equipped with a glassy carbon electrode cell and an Ag/AgCl reference cell. The working electrode was set at an applied potential of +1.0 V relative to an Ag/AgCl reference electrode, filter setting was 0.1 Hz, and range setting was 20 nA. The linear calibration curves were obtained and validated over the range of 10–1500 ng/mL in plasma with the quantitation limit of 10 ng/mL.

### 3.7. Lactate Production and Clearance during High-Intensity Swimming

The swimming test was carried out according to a pervious report [10] with slight modification. Rats were pretreated vehicle, RES-solution (50 mg/kg) and RES-SNEDDS (50 mg/kg), and, 6 h after administration, followed by lactate production and clearance test. The rats were individually placed in a water container (40 cm length, 40 cm width and 50 cm height) with 30-cm water depth maintained at 27 ± 1 °C. A weight equivalent to 5% of body weight was attached to the rat and a 10 min swimming test was performed. Blood samples were collected at pre-swimming, post-swimming and 30 min later after post-swimming. Blood lactate levels were analyzed using Lactate Pro™ 2 (LT-1730, Kyoto, Japan).

To realize the blood lactate production and clearance ratio during swimming test, a value of blood lactate production and clearance ratio were obtained using the following equation: Lactate production ratio = (Lactate_post-swimming_ − Lactate_pre-swimming_)/Lactate_pre-swimming_; Lactate clearance ratio = (Lactate_post-swimming_ − Lactate_30 min later after post-swimming_)/Lactate_post-swimming_.

### 3.8. Blood Biochemical Variables after Swimming

Plasma ammonia, CPK, and glucose were evaluated after swimming. After 6 h of administration, a weight equivalent to 5% of body weight was attached to the rat and a 15 min swimming test was performed. Blood samples were collected immediately from facial vein of rats. All of the blood biochemical levels were determined using an autoanalyzer (Beckman Coulter DXC800, Brea, CA, USA).

### 3.9. Exhaustive Swimming Test

After 6 h of administration, the rat was taken out from each treatment for exhaustive swimming test, and a weight equivalent to 15% of body weight was attached to the rat, as described previously [53]. Swimming time from the beginning to exhaustion was used to measure the endurance of each rat. The rats were considered exhausted when they loss of coordinated movements and failure to return to the surface within 7 s.

### 3.10. Tissue Glycogen Determination

Liver and skeletal muscles were used to investigate whether glycogen contents of these two tissues could enhance by RES-SNEDDS administration. Glycogen levels were measured using a glycogen colorimetric assay kit (BioVision, Milpitas, CA, USA). For each rat, 10 mg of liver and muscle was cut and weighed and then the tissues were rapidly homogenized with 100 µL ice cold glycogen hydrolysis buffer. After centrifugation at 12,000 rpm for 5 min at 4 °C, the supernatant was collected and added into a 96-well plate and brought the volume of 50 µL with glycogen hydrolysis buffer, following by adding 2 µL hydrolysis enzyme mix and 48 µL reaction mix containing glycogen development buffer, development enzyme mix and probe. After incubated at room temperature for 30 min, absorbance was read at 450 nm using a Varioskan Flash plate reader (Thermo Scientific, Waltham, MA, USA).

### 3.11. Statistical Analysis

Data are expressed as the mean ± standard error of the mean. Pharmacokinetic parameters were calculated by non-compartmental analysis [54]. PSS v14.0 (SPSS Inc., Chicago, IL, USA) was used to conduct the analysis of variance and the differences between formulations were compared using one-way analysis of variance followed by the least significant difference test. The *p* value less than 0.05 was regarded as significant.

## 4. Conclusions

An SNEDDS formulation for RES, which optimized self-emulsification ability, percentage transmittance, and particle size range, was developed. Pharmacokinetic studies in rats indicated that the developed RES-SNEDDS significantly (3.2-fold) improved the oral bioavailability of RES compared with RES in aqueous solution. Pretreatment with a single dose of the RES-SNEDDS before swimming exercise increased the swimming time to exhaustion of rats. In addition, RES-SNEDDS supplementation positively modulated exercise-induced fatigue-related parameters, namely lactate, ammonia, CPK, and glucose levels. Overall, our results indicated that the developed RES-SNEDDS improves RES absorption and protects against physical fatigue. This study first investigated the anti-fatigue effects of RES-SNEDDS in vivo by the enhancement of RES bioavailability and overcome the shortcomings of time-consuming pretreatment of pure RES. The designed SNEDDS efficiently enhanced the applicability of RES for improved oral absorption and anti-fatigue purpose, as evidenced by in vitro characterization, in vivo pharmacokinetics, and pharmacodynamics assessments.

## Figures and Tables

**Table 1:** Composition of resveratrol self-nanoemulsifying drug delivery system formulations and the results of dispersibility test, droplet size, polydispersity index (PDI) and percentage transmittance.

Formulation	Capryol 90/Cremophor EL/Tween 20 (%, *w*/*w*/*w*)	Dispersibility		Droplet Size (nm)	PDI	Transmittance (%)
		Water	0.1 N HCl			
F1	30:65:5	D	D	29.5 ± 1.8	0.91 ± 0.2	84.3 ± 0.3
F2	30:60:10	D	D	34.1 ± 10.9	1.10 ± 0.3	86.5 ± 1.0
F3	40:55:5	D	D	31.3 ± 6.8	0.98 ± 0.4	68.9 ± 0.7
F4	40:50:10	D	D	18.9 ± 5.6	1.15 ± 0.1	81.5 ± 1.4
F5	50:45:5	C	C	26.0 ± 3.3	0.84 ± 0.2	89.4 ± 1.4
F6	50:40:10	C	C	18.3 ± 6.6	1.86 ± 0.2	91.3 ± 0.3
F7	60:35:5	A	A	41.3 ± 4.1	0.38 ± 0.1	90.2 ± 0.5
F8	60:30:10	A	A	44.8 ± 11.9	1.00 ± 0.3	76.3 ± 0.6

Droplet size, PDI and percentage transmittance data are expressed as mean ± standard error of the mean (*n* = 3 for each group).

**Table 2:** Pharmacokinetic parameters of resveratrol (RES) after administration of RES self-nanoemulsifying drug delivery system (RES-SNEDDS) and RES-solution in rats.

Parameters	RES-SNEDDS (50 mg/kg, p.o.)	RES-Solution (50 mg/kg, p.o.)	RES-Solution (1 mg/kg, i.v.)
*T*_max_ (min)	42.0 ± 7.3	30.0	-
*C*_0_ or *C*_max_ (ng/mL)	869.2 ± 112.2 *	386.2 ± 68.4	3379.6 ± 431.7
*t*_1/2_ (min)	94.5 ± 11.1	78.9 ± 12.5	16.1 ± 3.8
AUC_0–t_ (ng min/mL)	77,055.4 ± 4857.7 *	23,950.5 ± 3691.3	16,215.8 ± 1892.5
Relative bioavailability (%)	321.7	-	-
Absolute bioavailability (%)	9.5	3.0	-

*T*_max_ is time of occurrence for maximum RES concentration, *C*_0_/*C*_max_ is maximum concentration of RES, *t*_1/2_ is RES half-life, AUC_0–t_ is RES area under the plasma concentration–time curve from zero (0) h to time (t). * Significantly different compared to RES-solution (50 mg/kg, p.o.) group (*p* < 0.05). Data are expressed as mean ± standard error of the mean (*n* = 5 for each group).
